# Supplementary material for: Identification of the fungal ligand triggering cytotoxic PRR-mediated NK cell killing of Cryptococcus and Candida
Source: Nat Commun. 2018 Feb 21;9:751. doi: 10.1038/s41467-018-03014-4 (PMC5821813; doi:10.1038/s41467-018-03014-4)
Supplement: Supplementary file 1 — Supplementary Information [file 41467_2018_3014_MOESM1_ESM.pdf]

## Supplemental Figures

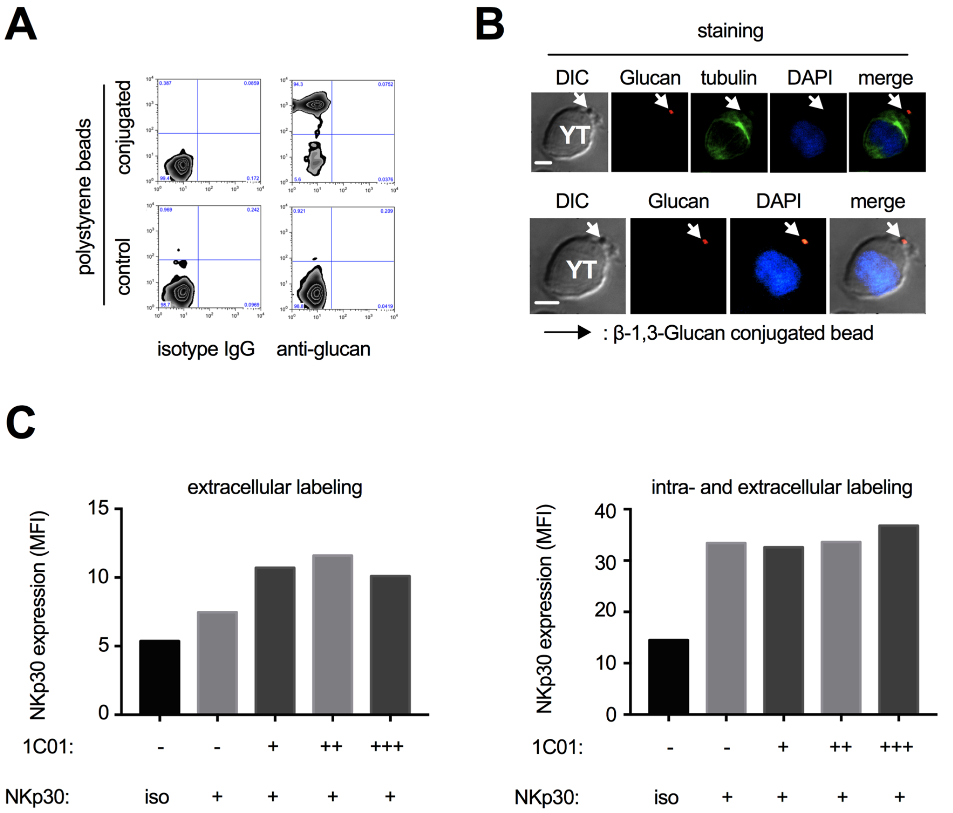


**Supplementary Figure 1**. Confirmation of the presence of β-1,3-glucan. (**A**) flow cytometric analysis of β-1,3-glucan on polystyrene beads (Guava EasyCyte™). All viable cells of interest as determined by forward and side scatter were included in the analysis. (**B**) microscopic detection of β-1,3-glucan on the polystyrene beads. For **A)** and **B**) the beads were conjugated with β-1,3-glucan as described [1]. (**C**) mAb 1C01 did not block binding of polyclonal antiNKp30. YT cells were treated with 1C01, washed and polyclonal anti-NKp30 antibody was used to label NKp30 on YT cells. Left panel, cell surface labeling on unpermeabilized cells; right panel, intracellular and cell surface labeling on permeabilized cells. Increasing concentrations of 1C01 were used for binding assay with similar results. All experiment measurement was performed for 3 times unless specified, and the results were graphed with the mean ± SEM.

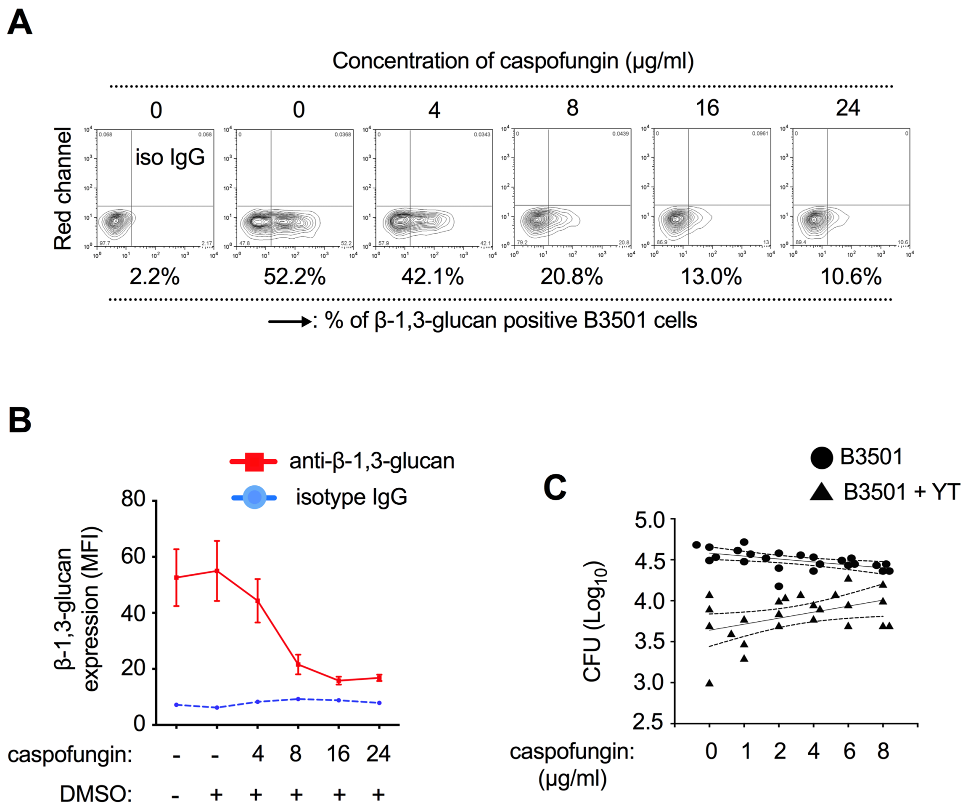


**Supplementary Figure 2**. Caspofungin disrupted β-1,3-glucan expression in *C. neoformans* (B3501). (**A**) representative flow cytometry analysis showed the % of β-1,3-glucan positive B3501 cells. All viable cells of interest as determined by forward and side scatter were included in the analysis. (**B**) mean fluorescent intensity of β-1,3-glucan expression on B3501 treated with various concentrations of caspofungin. All the experiments were repeated at least 3 times with similar results. B3501 was incubated with increasing concentrations of caspofungin for 5-6 hours. Expression of β-1,3-glucan on the treated B3501 was assessed with a monoclonal anti-β-1,3-glucan antibody (BioSupplies, Australia). (**C**) Treatment of *Cryptococcus* with caspofungin inhibits by YT cells. Mean CFU normalized by $Log_{10}$ were plotted against concentrations of caspofungin with regression analysis (95% confidence intervals) in the presence or absence of YT cells. Caspofungin was dissolved in DMSO, which was used as a control.

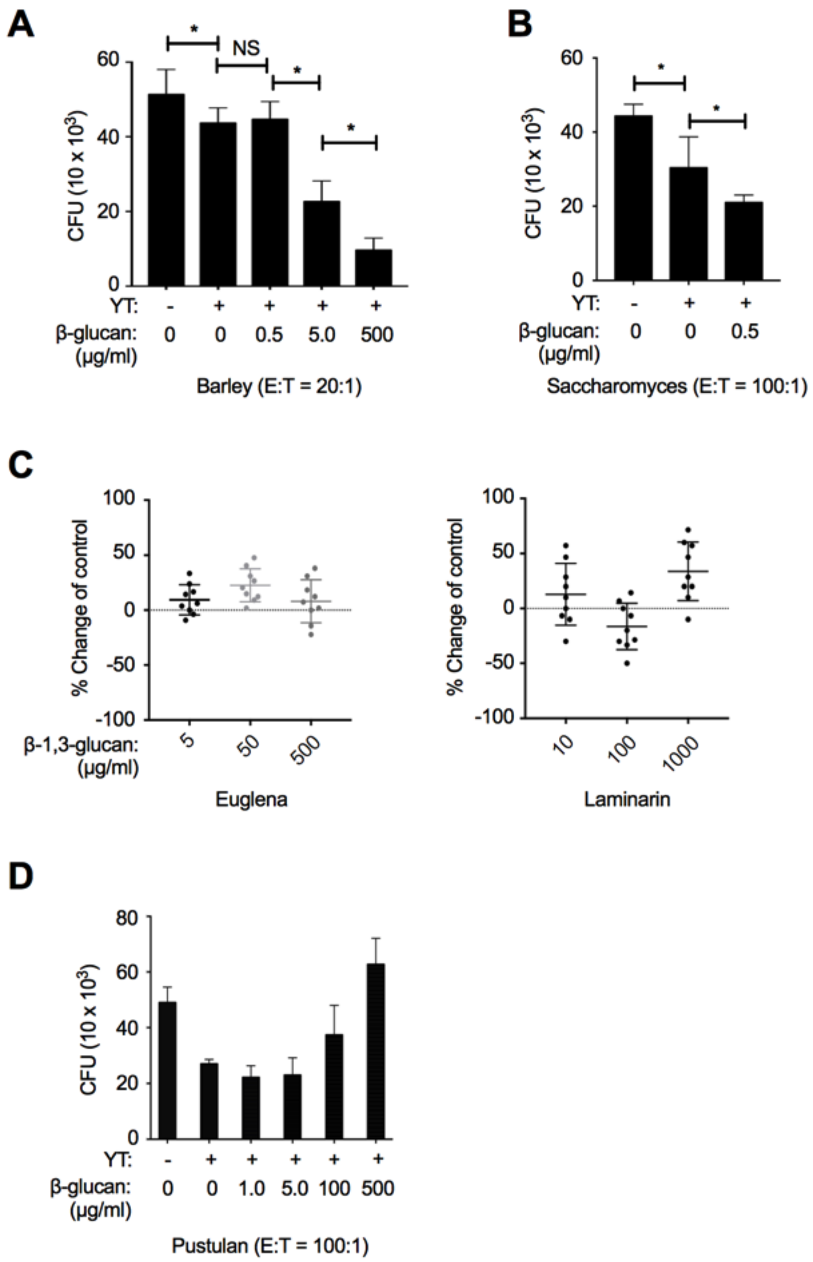


**Supplementary Figure 3**. β-1,3-glucan from various sources enhanced NK cell cytotoxicity. (**A**) β-1,3-glucan from barley enhanced YT cell killing of *Cryptococcus* (B3501). (**B**) β-1,3-glucan derived from *Saccharomyces cerevisiae* enhanced YT cell killing of *C. neoformans* (B3501). (**C**) the effect of β-1,3-glucan on cryptococcal growth. *C. neoformans* (strain B3501) was incubated with increasing amount of β-1,3-glucan as indicated at 37°C overnight, and then an aliquot was spotted onto agar plate as previously described [2]. All the experiments were repeated for 3 times with similar results. (**D**) effect of pustulan on YT cell killing of *C. neoformans* strain B3501. E:T, effector to target ratio; N.S.: non-significant; *, $p<0.05$.

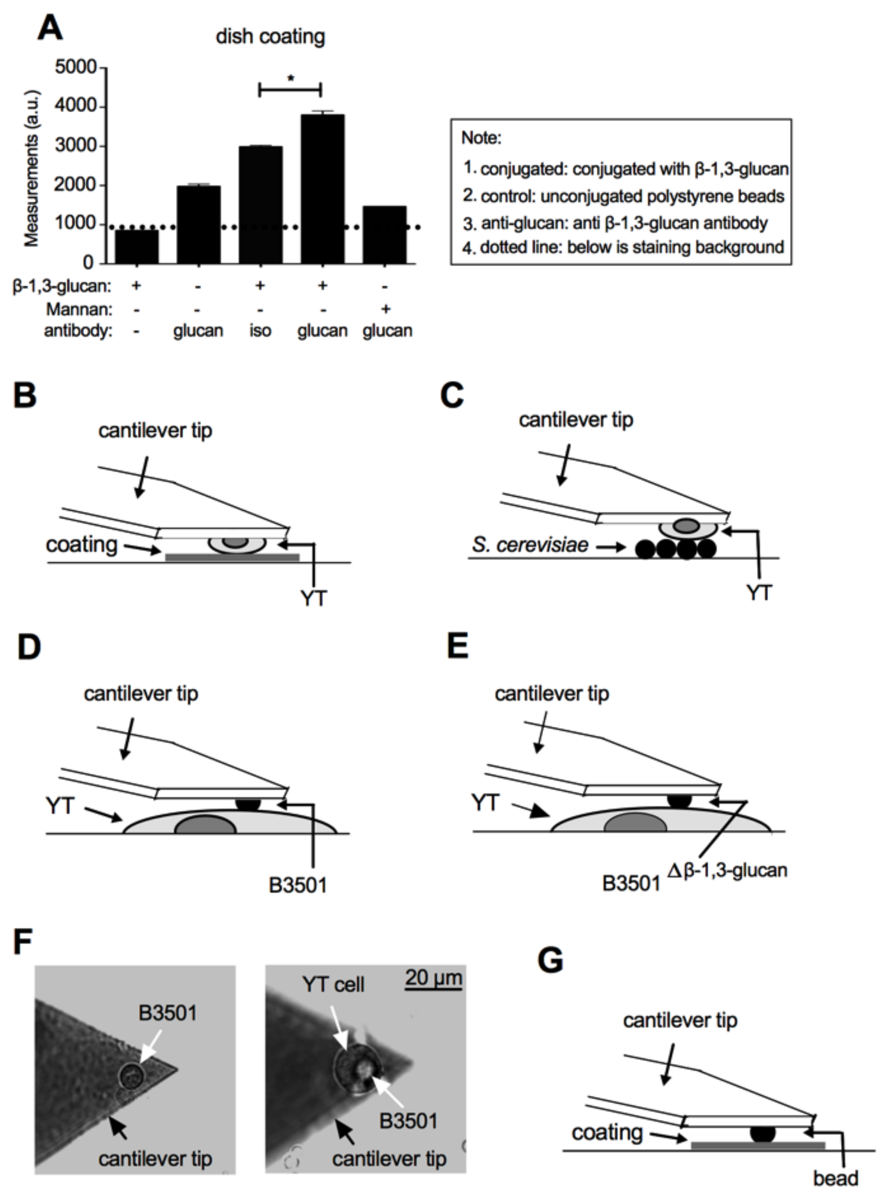


**Supplementary Figure 4. (A**) fluorescent illuminator was used to assess the presence of β-1,3-glucan on the glass. β-1,3-glucan was coated onto 0.1% poly-L-lysine-treated glass. 1,3-glucan was labeled with anti-β-1,3-glucan antibody (Biosupplies, Australia). (**B**-**G**) the configuration for SCFS measurements of binding force. **(B)** A single YT cell attached to a cantilever to β-1,3-glucan coating. **(C**) A single YT cell attached to a cantilever to *S. cerevisiae*. (**D**) Binding of untreated *C. neoformans* (B3501) to a single YT cell. (**E**) Binding of caspofungin-treated B3501 to a single YT cell. (**F**) a representative photomicrograph demonstrating a single B3501 bound to a single YT cell. (**G**) binding of a conjugated or unconjugated beads on cantilever to recombinant NKp30 bound to glass.

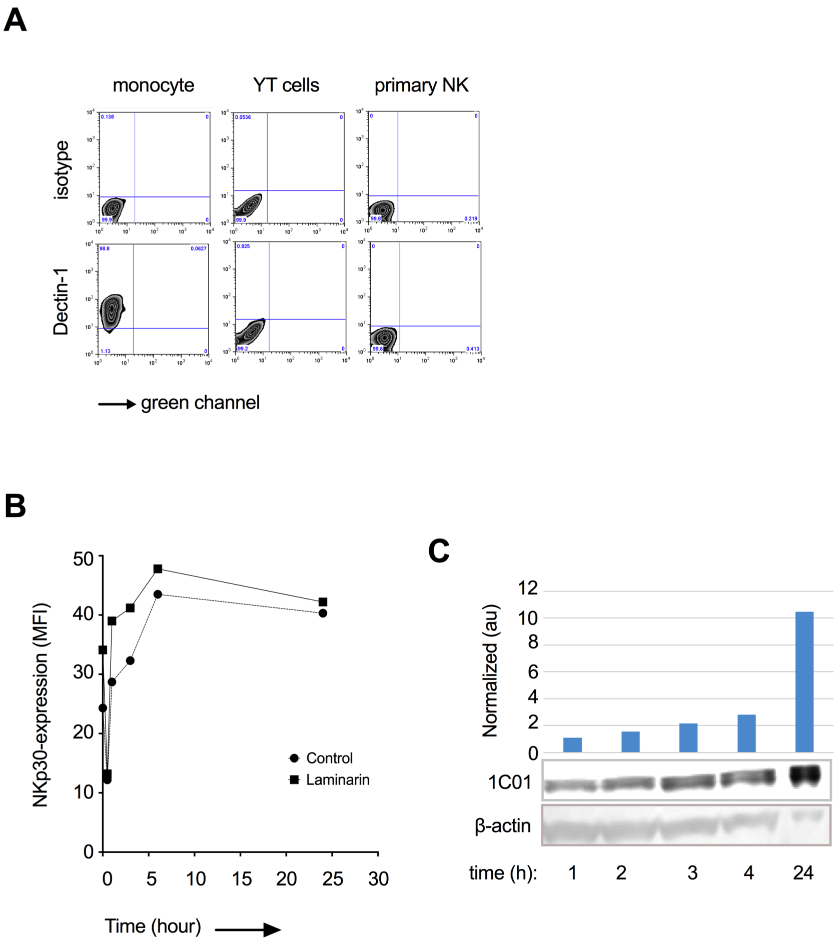


**Supplementary Figure 5**. (**A**) detection of dectin-1 on monocytes, primary NK cells and YT cells using flow cytometry. (**B**) Flow cytometric analysis of NKp30 expression stimulated by β-1,3-glucan. (**C**) Western blot analysis of NKp30 expression stimulated by β-1,3-glucan. YT cells were mixed with β-1,3-glucan (laminarin) and incubated at 37°C for the indicated times. The bars represent the intensity of the bands normalized for the β-actin control in arbitrary units (au). 1C01 was used and IgG2a as control. All viable cells of interest analyzed by flow cytometry as determined by forward and side scatter were included in the analysis. All the experiments were repeated for at least 3 times with similar results.

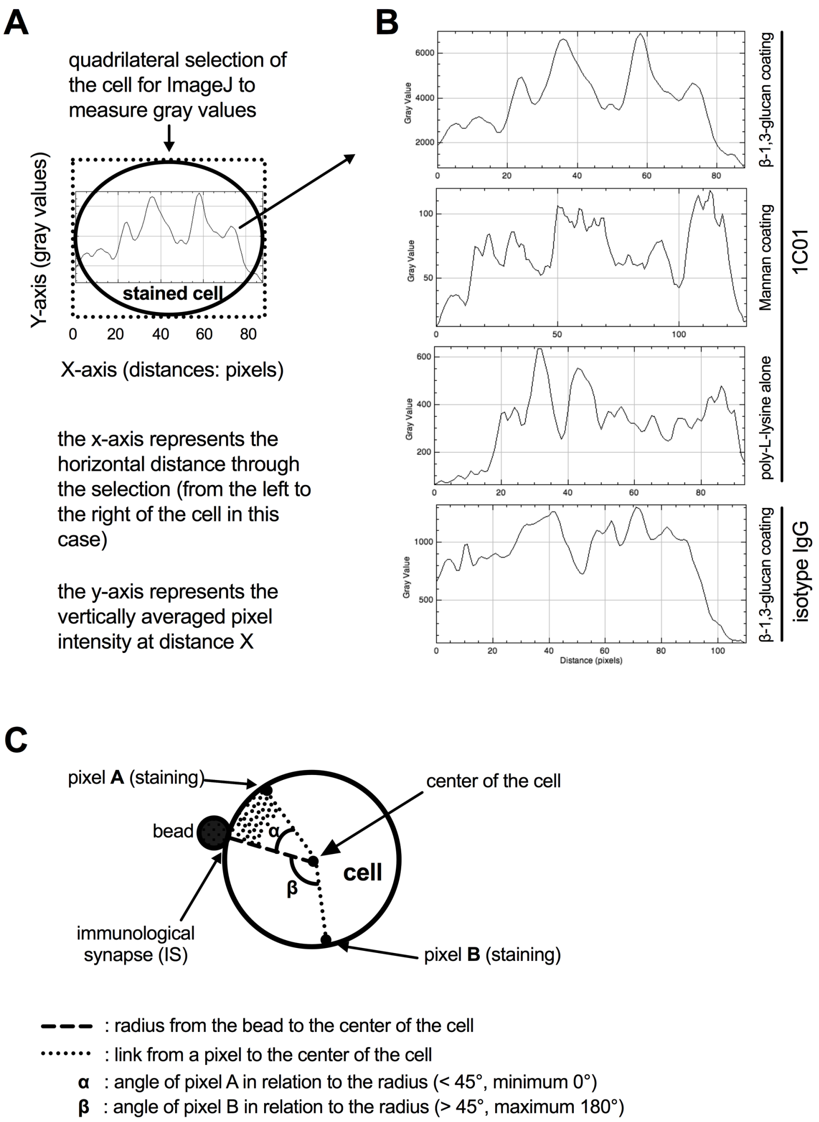


**Supplementary Figure 6. (A)** Methodology for TIRF quantification of NKp30 staining in YT cells adhered to matrices by ImageJ (NIH). The labeling intensity profile of 1C01 or isotype control IgG was performed with ImageJ as described in Materials and Methods. **(B)** Quantification of representative 1C01 labeling of YT cells adhered to β-1,3-glucan and controls. Poly-L-lysine alone and mannan coating were used as control matrix. **(C)** schematic drawing of methodologies to quantify and analyze NKp30 labeling in YT cells in response to polystyrene beads conjugated with β-1,3-glucan. Isotype IgG2a was used as control for 1C01. The vertical scale for each condition is different.

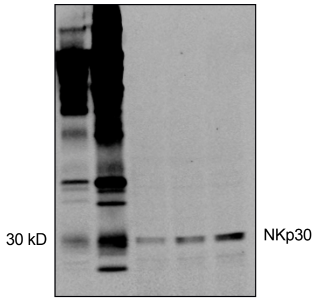


original WB blot for Figure 1H

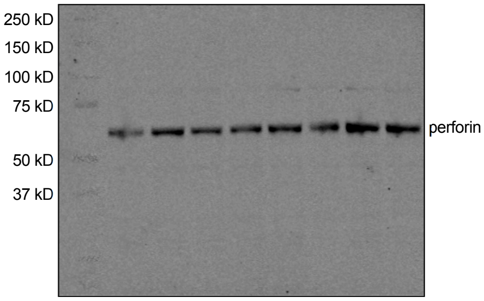


original WB blot for Figure 4G

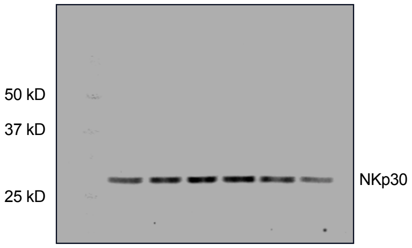


original WB blot for Figure 5B

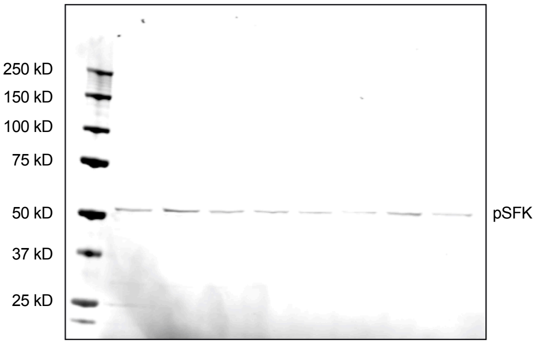


original WB blot for Figure 6A

**Supplementary Figure 7.** Full Western blots for Figure 1H, 5B and 6A.

## Supplemental References


1. Tam JM, Mansour MK, Khan NS, Yoder NC, Vyas JM. Use of fungal derived polysaccharide-conjugated particles to probe Dectin-1 responses in innate immunity. *Integr Biol (Camb)* **4**, 220-227 (2012).

2. Li SS*, et al.* The NK receptor NKp30 mediates direct fungal recognition and killing and is diminished in NK cells from HIV-infected patients. *Cell host & microbe* **14**, 387-397 (2013).
