# Supplementary material for: Identification of the fungal ligand triggering cytotoxic PRR-mediated NK cell killing of Cryptococcus and Candida
Source: Nat Commun. 2018 Feb 21;9:751. doi: 10.1038/s41467-018-03014-4 (PMC5821813; doi:10.1038/s41467-018-03014-4)
Supplement: Supplementary file 3 — Description of Additional Supplementary Files [file 41467_2018_3014_MOESM3_ESM.pdf]

Description of Additional Supplementary Files

File Name: Supplementary Movie 1
Description: Granule polarization in YT cell response to beads conjugated with β-1,3- glucan in the presence of Lysotracker® Red. Total time is 2 hours from the start to the end of the recording (20 frames/sec, 5 sec time lapse, 1 sec in video equals 1 min 40 sec in real time. Images from this video are displayed in Figure 4.

File Name: Supplementary Movie 2
Description: Granule polarization in YT cell response to unconjugated beads in the presence of Lysotracker® Red. Total time: 2 h 7 min from the start to the end of the recording (20 frames/sec, 5 sec time lapse, 1 sec of the video is equivalent of 1 min 40 sec). Images from this video are displayed in Figure 4.

File Name: Supplementary Movie 3
Description: Granule polarization in YT cell response to beads conjugated with mannan in the presence of Lysotracker® Red. Total time: 2 h 40 min from the start to the end of the recording (20 frames/sec, 15 sec time lapse, 1 sec in the video equals 5 min in actual recording time).

File Name: Supplementary Movie 4
Description: NKp30 is required granule polarization in YT cells in response to beads conjugated with β-1,3-glucan. YT cells were labeled with Lysotracker® Red and treated with 25 ul (12.5 μg/ml) 1C01 for 30 min before beads were added. The length of the whole movie from the start to the end of recording is 2 h 32 min (5 frames/sec, 15 sec time lapse, 1 sec in the video equals 1 min 15 sec in actual recording time). Images from this video are displayed in Figure 6.

File Name: Supplementary Movie 5
Description: SFK is required for granule polarization in YT cells in response to beads conjugated with β-1,3-glucan. YT cells were labeled with Lysotracker® Red and treated with 50 nM dasatinib for 30 min before beads were added. The length of the whole movie is 1 h 6 min (5 frames/ sec, 15 sec time lapse, 1 sec in the video equals 1 min 15 sec in actual recording time). Images from this video are displayed in Figure 6.

File Name: Supplementary Movie 6
Description: Effect of isotype IgG (control for 1C01) on granule polarization in YT cell responding to beads conjugated with β-1,3-glucan. YT cells were treated with 0.2 μl/ml (equivalent amount of 1C01) of isotype IgG for 1C01 for 30 min before beads were added. The length of the whole movie is 1 h 20 min (5 frames/sec, 15 sec time lapse, 1 sec in the video equals 1 min 15 sec in actual recording time). Images from this video are displayed in Figure 6.

File Name: Supplementary Movie 7
Description: Granule polarization in YT cells that had NKp30 knocked down in response to beads conjugated with β-1,3-glucan. Lysotracker® Red was used to label granules. Total time is 84 min from the start to the end of the recording (20 frames/sec, 5 sec time lapse, 1 sec in video equals 7 min 30 sec in real time.
